# Can visceral adipose tissue and skeletal muscle predict recurrence of newly diagnosed Crohn’s disease in different treatments

**DOI:** 10.1186/s12876-022-02327-5

**Published:** 2022-05-18

**Authors:** Zinan Zhang, Xiaoyu Yu, Ning Fang, Xiuyan Long, Xixian Ruan, Jianing Qiu, Sifan Tao, Pan Gong, Kai Nie, An Li, Xiaoyan Wang, Li Tian

**Affiliations:** Department of Gastroenterology, The Third Xiangya Hospital of Central South University, 138 Tongzipo Road, Changsha, 410013 Hunan China

**Keywords:** Visceral adipose tissue, Skeletal muscle, Crohn’s disease

## Abstract

**Background and aims:**

It is crucial to manage the recurrence of Crohn’s disease (CD). This study is aimed to explore whether visceral adipose tissue (VAT) and skeletal muscle (SM) are associated with the recurrence of CD upon different treatments.

**Methods:**

All patients with a definite diagnosis of CD were retrospectively divided into three groups according to distinct treatment regimens: 5-amino salicylic acid group (Group A), steroids + azathioprine (Group B) and biologics (Group C). The pretreatment computerized tomography (CT) images and clinical data were collected. The VAT area, mesenteric fat index (MFI), the ratio of VAT area to fat mass (VAT area/FM) were assessed. The primary end point was the recurrence of CD within 1 year of follow-up.

**Results:**

A total of 171 CD patients were enrolled, including 57 (33.33%) patients in Group A, 70 (40.94%) patients in Group B and 44 (25.73%) patients in Group C. Patients with 1-year recurrence had higher MFI (*P* = 0.011) and VAT area/FM (*P* = 0.000). ROC curve demonstrated that patients with the ratio of VAT area/FM and MFI higher than 0.578 and 1.394 tended to have recurrence with the AUC of 0.707 and 0.709. Similar results could be observed in Group A & B but not in Group C.

**Conclusions:**

High VAT area/FM and MFI are related to recurrence within 1 year for newly diagnosed CD patients treated by 5-amino salicylic or azathioprine + steroids rather than biologics. We could not observe any radiological data associated with the recurrence of CD patients under biological treatment.

## Introduction

Crohn's disease (CD) is a progressive and disabling gastrointestinal inflammatory disease with a broad spectrum of clinical manifestations, commonly occurs in young adults. In recent years, the number of CD patients has been increasing rapidly [1]. CD has the characteristics of incurability and lifelong recurrence. With repeated relapses, patients have a high demand for surgery. More than half of the postoperative patients have a clinical recurrence of 10 years [2]. In addition, plenty of them suffered from postoperative complications such as short bowel syndrome and postoperative anastomotic leakage [3]. Therefore, preventing or delaying disease relapse is critical to CD management.

According to the Chinese CD guideline, the treatments for CD including 5-amino salicylic acid, corticosteroids plus immunosuppressants, and biologics are selected primarily based on the severity of the disease and the response to the treatment [4]. Early use of biologics is considered to be the most beneficial treatment [5–7]. However, the high cost, loss of response and potential infection risk limit the application of biologics. Hence, predicting the patients’ response to the treatments and practicing precise medicine on CD patients is important.

There are many prognostic factors affecting treatment outcomes in CD [8]. Visceral adipose tissue (VAT) and skeletal muscle (SM) are novel factors that can predict the outcome reliably in other diseases [9–11]. Previous studies demonstrated that VAT and SM were related to CD-associated lesions including penetrating lesions, stricturing lesion, and perianal diseases [12, 13]. Thus, VAT is a possible relapsing predictive factor. Although certain studies found that VAT was associated with adverse outcomes including reoperation, death within 2 years, or endoscopic recurrence, the different impact based on various treatments was not considered comprehensively [12, 14, 15]. Moreover, the existence of VAT has been proven to change the therapeutic effect of biologics [16]. To date, there are no studies focusing on the association between CD recurrence and VAT in different treatments. We conduct a retrospective study to verify the hypothesis that VAT is associated with the recurrence of CD in different treatments.

## Methods

### Patients

This retrospective study was approved by the Ethical Committee of the Third Xiangya Hospital of Central South University, China (IRB No. R19057). Since this study retrospectively collected the existing medical records of our center, the Ethical Committee of the Third Xiangya Hospital of Central South University approved the requirement for informed consent could be waived. All the treatment for the patients were performed in accordance with the relevant guidelines and regulations.

#### Study design

**Fig. 1:**
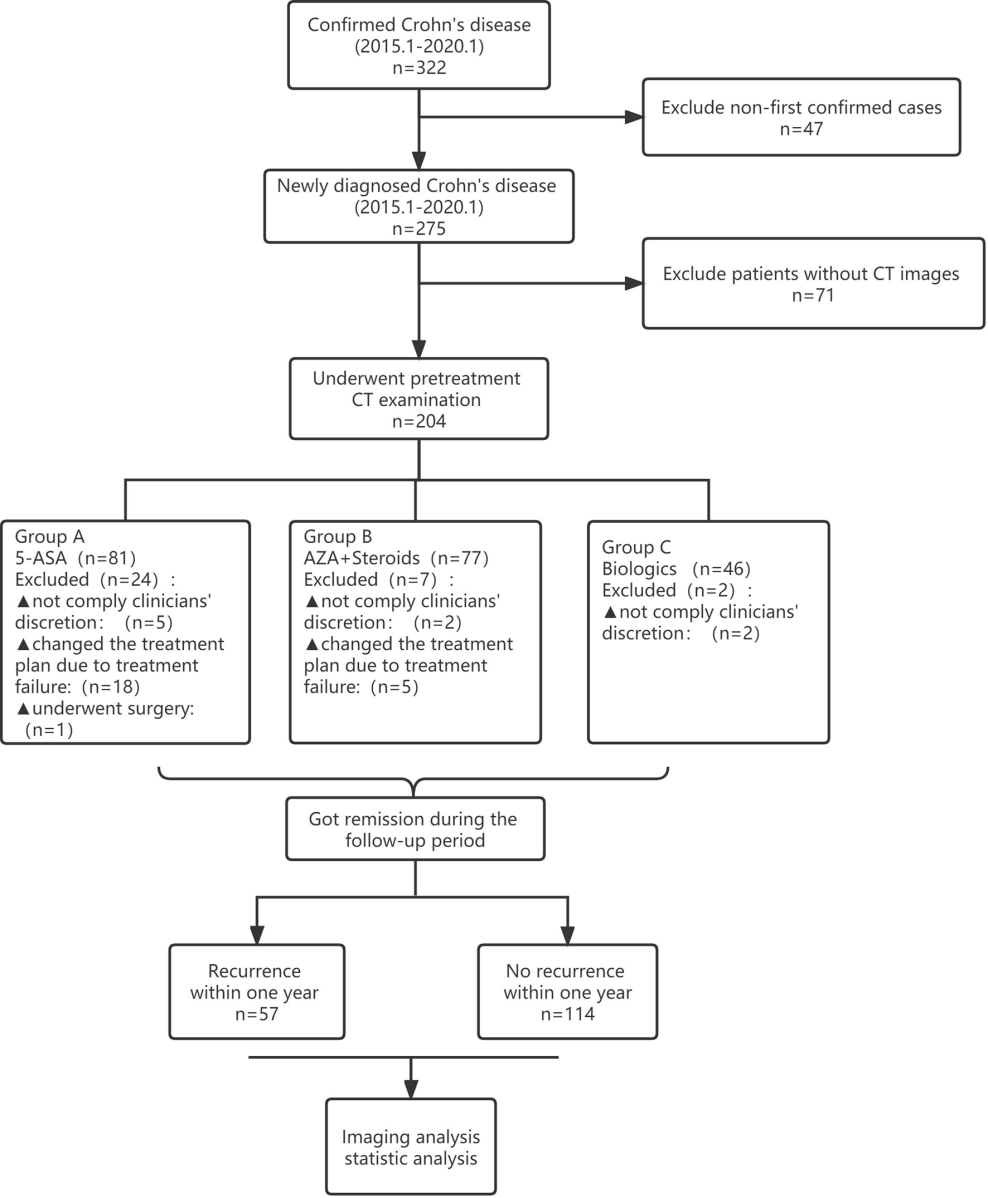
Inclusion and exclusion process of participants

Consecutive data of patients who were first diagnosed with active Crohn’s disease in our center from January 2015 to January 2020 was retrospectively collected in the present study. Patients were treated with Anti-infective and Symptomatic treatment before their were diagnosed with active Crohn’s disease. Patients who realized remission after the primary treatment based on the result of the follow-up were enrolled in this study. Those patients who failed to have remission were considered as treatment failure or non-response and excluded. Patients without CT data or treated by surgery was also excluded. The enrollment process of this study cohort, including inclusion and exclusion criteria, is demonstrated in Fig. 1.

All enrolled participants were divided into 3 groups including 5-amino salicylic acid (4 g/d) group (Group A), corticosteroids (0.75–1.0 mg/kg) and immunosuppressants (1.5–2.0 mg/kg) (Group B), and biologics (5 mg/kg) (Group C) based on their primary treatment. Patients in group B were treated with azathioprine for maintenance therapy after induction of remission with 0.75–1.0 mg/kg steroids for a short period of time. Corticosteroids gradually decreased after 2 to 4 weeks and would be stopped in 3 to 4 months. All patients' treatment plans are based on Chinese CD guidelines, using a joint doctor-patient decision-making approach to select treatment options that patients and their families can afford or desire. All the enrolled patients used concomitant treatments such as protecting the intestines and regulating intestinal flora according to their own conditions at different periods of their disease course.

#### Follow-up

The follow-up period included clinical disease activity index (CDAI) assessment, erythrocyte sedimentation rate (ESR) test, C-reactive protein (CRP) test and SES-CD assessment for each patient exceeded 1 year. The CDAI, ESR, CRP and SES-CD were performed 3–4 months after the primary treatment. If the patient got remission, they would be reevaluated through CDAI, SES-CD and improvement of clinical symptoms 9–12 months after the primary treatment.

### Study outcome

The primary outcome was recurrence within 1 year after the patients received clinical remission. The following two conditions were considered to be CD recurrence. (1) The patients got CDAI ≥ 150 [17] or simplified endoscopic activity score for Crohn’s disease (SES-CD) [18] ≥ 2 during the follow-up period within 1 year. (2) Any CD-related hospitalization, surgery, need for rescue corticosteroids, and treatment dose escalation within 1 year [4, 19, 20]. As long as one of the above conditions occurred, the patient was considered to have recurrence. The treatment and follow-up results after the recurrence will not be included in the statistical analysis.

### Computed tomography image analysis

**Fig. 2:**
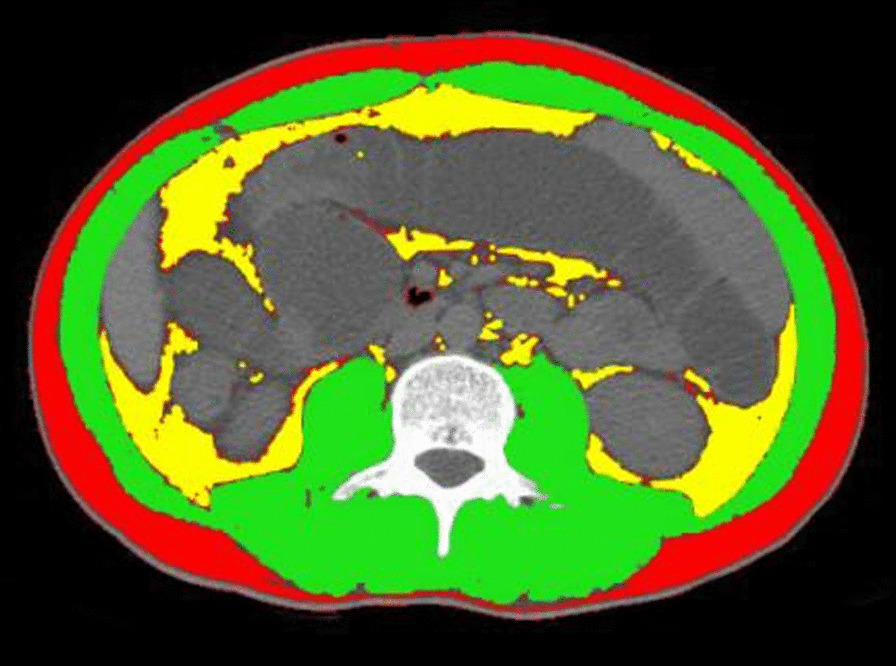
Evaluation of the body composition using a third lumbar computed tomography scan slice. Red: subcutaneous adipose tissue (SAT). Green: skeletal muscle (SM). Yellow: visceral adipose tissue (VAT)

Computed tomography (CT) images of patients at the level of the third vertebra (L3) were obtained for VAT, subcutaneous adipose tissue (SAT), fat mass (FM) and skeletal muscle (SM) measurement. If abdominal CT examinations were performed repeatedly, only the first CT examination performed after admission would be considered. Each selected image was assessed by a single reviewer blinded to clinical and biological data using the free public-domain software developed by the National Institutes of Health (NIH Image J1.47). The VAT area (cm^2^) and SAT (cm^2^) area were outlined and measured using standard threshold values of − 150 to − 50 Hounsfield unit (HU). The SM area (cm^2^) was outlined and calculated using standard threshold values of − 30 to + 150 HU (Fig. 2). As reported before in the literature, the visceral obesity was defined as VAT area ≥ 130 cm^2^ [21]. Mesenteric fat index (MFI) (cm^2^/cm^2^), also known as the visceral fat index (VFI), was defined as the ratio of VAT to SAT. The skeletal muscle index (SMI) (cm^2^/m^2^) was the ratio of the SM to height squared (m^2^). Men with lower than 52.4 cm^2^/m^2^ SMI and women with lower than 38.5 cm^2^/m^2^ SMI were diagnosed with sarcopenia [22].

### Statistical analysis

The recorded data were analyzed by IBM SPSS version 23.0 (IBM Corporation, Armonk, NY, USA). The Chi-square test or Fisher's precision probability test was used to compare categorical variables, and the Student’s t-test or Mann–Whitney and Wilcoxon tests were used for quantitative variables. The receiver operating characteristic (ROC) curve was drawn to evaluate the effectiveness of a variable to distinguish whether patients would meet recurrence. The least absolute shrinkage and selection operator (LASSO) was conducted to evaluate the coefficients of all enrolled factors related to the relapse. We used LASSO to avoid potential collinearity between factors for better validity of the results. *P*-value < 0.05 was considered statistically significant.

## Results

A total of 171 CD patients were enrolled in this study including 57 (33.33%) patients in Group A, 70 (40.94%) patients in Group B and 44 (25.73%) patients in Group C. Among all patients, a total of 57 (33.33%) had CD recurrence within 1 year, and 114 (66.67%) did not. There was no statistical difference in all the demographic and disease characteristics among all groups (Table 1).

**Table 1:** The demographic and disease characteristics information of all patients

Characteristics	Group A			Group B			Group C			Overall		
	Relapse 29 (50.88%)	Not Relapse 28 (49.12%)	*P*	Relapse 22 (31.43%)	Not Relapse 48 (68.57%)	*P*	Relapse 6 (13.64%)	Not Relapse 38 (86.36%)	*P*	Relapse 57 (33.33%)	Not Relapse 114 (66.67%)	*P*
Age*, mean ± sd	39.07 ± 13.72	37.18 ± 13.69	0.605	37.00 ± 15.49	34.21 ± 12.36	0.421	29.17 ± 9.80	29.58 ± 13.20	0.942	38.81 ± 16.72	33.39 ± 13.20	0.083
Gender, n (%)												
Male	25 (86.21%)	20 (71.43%)	0.207	20 (90.90%)	38 (79.17%)	0.195	4 (66.67%)	32 (84.21%)	0.297	49 (85.96%)	90 (78.95%)	0.267
Female	4 (13.80%)	8 (28.57%)		2 (9.91%)	10 (20.83%)		2 (33.33%)	6 (15.79%)		8 (14.04%)	24 (21.05%)	
Duration of Symptoms, median(interquartile range), (month)	6.00(22.33)	10.00(21.33)	0.501	36.50(47.83)	12.17(31.75)	0.062	30.41(112.04)	12.17(31.00)	0.256	36.5(47.83)	12.17(31.75)	0.407
¾Location**, n (%)												
L1	13 (44.83%)	14 (50.00%)	0.493	12 (54.53%)	21 (43.75%)	0.267	2 (33.33%)	18 (47.37%)	0.785	27 (47.37%)	53 (46.49%)	0.993
L2	4 (13.79%)	1 (3.57%)		2 (9.91%)	8 (16.67%)		0 (0.00%)	3 (7.89%)		6 (10.53%)	12 (10.53%)	
L3	12 (41.38%)	13 (46.43%)		8 (36.36%)	19 (39.58%)		4 (66.67%)	17 (44.74%)		24 (42.11%)	49 (42.98%)	
Behaviour**,n (%)												
B1	21 (72.41%)	22 (78.57%)	0.871	15 (68.18%)	39 (81.25%)	0.369	3 (50.00%)	29 (76.32%)	0.106	39 (68.42%)	90 (78.95%)	0.602
B2	1 (3.45%)	1 (3.57%)		4 (18.18%)	4 (8.33%)		1 (16.67%)	7 (18.42%)		6 (10.53%)	12(10.53%)	
B3	7 (24.14%)	5 (17.86%)		3 (13.64%)	5 (10.42%)		2 (33.33%)	2 (5.26%)		12 (21.05%)	12(10.53%)	
Perianal disease, n (%)												
Yes	5 (17.24%)	3 (10.71%)	0.706	7 (31.82%)	9 (18.75%)	0.182	0 (0.00%)	8 (21.05%)	0.276	12 (21.05%)	20 (17.54%)	0.579
No	24 (82.76%)	25 (89.29%)		15 (68.18%)	39 (81.25%)		6 (100.00%)	30 (78.95%)		45 (78.95%)	94 (82.46%)	
CDAI	336.41 ± 102.95	317.18 ± 85.22	0.446	322.45 ± 97.66	350.50 ± 123.01	0.310	355.33 ± 97.66	332.21 ± 103.71	0.605	340.44 ± 103.59	329.17 ± 101.09	0.496
SES-CD	5.90 ± 2.51	5.70 ± 2.08	0.752	6.32 ± 1.96	6.67 ± 2.10	0.513	4.33 ± 3.39	5.97 ± 2.35	0.143	5.92 ± 2.37	6.20 ± 2.20	0.446
Previous treatment (%)												
Anti-infective	3 (10.34%)	1 (3.57%)	0.299	2 (9.09%)	4 (8.33%)	0.486	0 (0.00%)	2 (5.26%)	0.531	5 (8.77%)	7 (6.14%)	0.104
Symptomatic treatment***	8 (27.59%)	14 (50.00%)		10 (45.45%)	21 (43.75%)		1 (16.67%)	16 (42.11%)		19 (33.33%)	51 (44.74%)	
Anti-infective and Symptomatic treatment	8 (27.59%)	4 (14.29%)		3 (13.64%)	2 (4.17%)		1 (16.67%)	4 (10.53%)		12 (21.05%)	10 (8.77%)	
None	10 (34.48%)	9 (32.14%)		7 (31.82%)	21 (43.75%)		4 (66.67%)	16 (42.11%)		21 (36.84%)	46 (40.35%)	

*Age collected for data analysis refers to the patient’s age when he or she was first diagnosed with Crohn's disease

**According to the Montreal classification: *L1* ileal, *L2* colonic, *L3* ileocolonic, *B1* non-stricturing non-penetrating, B2 stricturing, *B3* penetrating

***Symptomatic treatment refers to protecting the intestines, regulating intestinal flora, etc.

For all patients with and without 1-year recurrence, the mean VAT area was 54.63 ± 42.50 VS 44.63 ± 42.99, mean SAT was 49.68 ± 38.11 VS 57.93 ± 41.28, mean MFI was 1.42 ± 0.83 VS 0.99 ± 1.10 and mean VAT/FM was 0.53 ± 0.14VS 0.42 ± 0.16. One-year relapsed patients had higher MFI (*P* = 0.011) and VAT/FM (*P* = 0.000). Although there was no statistical difference in SM and SMI, patients who had sarcopenia defined based on the SM and SMI were more likely to relapse within 1 year (*P* = 0.022). A total of 1 (1.75%) relapsed patient who relapsed and 14 (12.28%) non-relapsed patients who were not diagnosed with sarcopenia. For biological data, only albumin was related to the recurrence within 1 year. The mean albumin was 32.77 ± 6.59 VS 34.97 ± 5.67 in patients with and without recurrence (*P* = 0.028, Table 2).

**Table 2:** The radiological, anthropometric and biological data overall and in all groups

Characteristics	Group A			Group B			Group C			Overall		
	Relapse 29 (50.88%)	Not Relapse 28 (49.12%)	*P*	Relapse 22 (31.43%)	Not Relapse 48 (68.57%)	*P*	Relapse 6 (13.64%)	Not Relapse 38 (86.36%)	*P*	Relapse 57 (33.33%)	Not Relapse 114 (66.67%)	*P*
*Radiological and Anthropometric data*												
VAT, mean ± sd	51.94 ± 45.52	48.83 ± 43.54	0.495	59.78 ± 42.28	56.73 ± 47.64	0.798	41.20 ± 13.20	31.14 ± 33.22	0.471	54.63 ± 42.50	44.63 ± 42.99	0.152
SAT, mean ± sd	44.81 ± 46.49	66.44 ± 43.89	0.147	48.83 ± 25.64	63.80 ± 39.65	0.110	57.02 ± 36.49	44.25 ± 38.87	0.455	49.68 ± 38.11	57.93 ± 41.28	0.208
MFI, mean ± sd	1.51 ± 0.88	0.63 ± 0.38	0.000	1.32 ± 0.62	1.04 ± 0.97	0.210	1.32 ± 1.32	1.20 ± 1.50	0.858	1.42 ± 0.83	0.99 ± 1.10	0.011
VAT/FM, mean ± sd	0.55 ± 0.13	0.35 ± 0.13	0.000	0.53 ± 0.14	0.44 ± 0.15	0.030	0.46 ± 0.22	0.44 ± 0.18	0.810	0.53 ± 0.14	0.42 ± 0.16	0.000
Visceral obesity, n (%)												
Yes	2 (6.90%)	1 (3.58%)	1.000	1 (4.55%)	4 (8.33%)	0.495	0 (0.00%)	1 (2.63%)	0.864	3 (5.26%)	6 (5.26%)	1.000
No	27 (93.10%)	27 (96.42%)		21 (95.45%)	44 (91.67%)		6 (100.00%)	37 (97.37%)		54(94.74%)	108 (94.74%)	
SM, mean ± sd	120.58 ± 25.08	120.08 ± 26.88	0.942	118.05 ± 27.32	115.97 ± 26.85	0.765	112.48 ± 26.80	121.90 ± 27.50	0.438	118.75 ± 25.78	118.95 ± 26.96	0.963
SMI, mean ± sd	42.32 ± 6.55	43.08 ± 8.18	0.699	41.05 ± 8.10	41.76 ± 7.75	0.728	40.19 ± 8.09	42.63 ± 8.15	0.498	41.60 ± 7.25	42.37 ± 7.94	0.540
Sarcopenia, n (%)												
Yes	29 (100.00%)	25 (89.29%)	0.112	21 (95.45%)	42 (87.50%)	0.287	6 (100.00%)	33 (86.84%)	0.462	56 (98.25%)	100 (87.72%)	0.022
No	0 (0.00%)	3 (10.71%)		1 (4.55%)	6 (12.50%)		0 (0.00%)	5 (13.16%)		1 (1.75%)	14 (12.28%)	
BMI*, mean ± sd	18.83 ± 2.65	19.50 ± 2.94	0.370	18.81 ± 2.83	19.24 ± 2.61	0.532	18.85 ± 2.21	18.69 ± 2.80	0.900	18.83 ± 2.64	19.12 ± 2.75	0.498
Standard body weight												
Underweight	15 (51.72%)	13 (46.43%)	0.290	11 (50.00%)	22 (45.83%)	1.000	2 (33.33%)	18 (47.37%)	0.751	28 (49.12%)	53 (46.49%)	0.180
Normal BMI	14 (48.28%)	12 (42.86%)		11 (50.00%)	24 (50.00%)		4 (66.67%)	18 (47.37%)		29 (50.88%)	54 (47.37%)	
Overweight	0 (0.00%)	3 (10.71%)		0 (0.00%)	2 (4.17%)		0 (0.00%)	2 (5.26%)		0 (0.00%)	7 (6.14%)	
*Biological data*												
ESR, mean ± sd	37.89 ± 28.71	27.41 ± 34.02	0.227	36.35 ± 31.51	47.46 ± 35.83	0.233	29.83 ± 13.36	45.89 ± 34.99	0.062	36.40 ± 28.25	42.22 ± 35.90	0.264
High ESR, n (%)												
Yes	17 (62.97%)	9 (34.62%)	0.039	13 (65.00%)	37 (74.00%)	0.231	5 (83.33%)	24 (70.59%)	0.464	35 (66.04%)	70 (64.81%)	0.878
No	10 (37.04%)	17 (65.38%)		7 (35.00%)	11 (26.00%)		1 (16.67%)	10 (29.41%)		18 (33.96%)	38 (35.19%)	
CRP, mean ± sd	42.75 ± 65.49	20.78 ± 43.23	0.167	25.86 ± 28.15	29.34 ± 34.81	0.706	42.01 ± 44.07	39.00 ± 53.50	0.897	36.82 ± 52.82	30.66 ± 43.77	0.437
High CRP, n (%)												
Yes	25 (89.29%)	12 (50.00%)	0.002	11 (61.11%)	34 (70.83%)	0.319	5 (83.33%)	26 (72.22%)	0.497	41 (78.85%)	72 (66.67%)	0.113
No	3 (10.71%)	12 (50.00%)		7 (38.89%)	14 (29.17%)		1 (16.67%)	10 (27.78%)		11 (21.15%)	36 (33.33%)	
Platelet counts, mean ± sd	345.03 ± 179.27	289.79 ± 106.09	0.162	309.00 ± 124.37	348.35 ± 143.18	0.288	363.33 ± 99.92	333.16 ± 115.17	0.548	333.93 ± 153.05	328.90 ± 126.91	0.822
High platelet counts, n (%)												
Yes	27 (93.10%)	26 (92.86%)	1.000	18 (90.00%)	47 (97.92%)	0.205	6 (100.00%)	35 (92.11%)	0.637	51 (92.73%)	108 (97.74%)	0.730
No	2 (6.90%)	2 (7.14%)		2 (10.00%)	1 (2.08%)		0 (0.00%)	3 (7.89%)		4 (7.27%)	6 (5.26%)	
Albumin**, mean ± sd	32.20 ± 6.49	35.74 ± 5.46	0.033	34.16 ± 6.42	35.22 ± 6.45	0.536	30.70 ± 7.86	34.08 ± 4.69	0.144	32.77 ± 6.59	34.97 ± 5.67	0.028
Low albumin, n (%)												
Yes	25 (92.59%)	21 (75.00%)	0.143	15 (75.00%)	38 (79.19%)	0.467	6 (100.00%)	33 (86.84%)	0.462	46 (86.79%)	92 (80.70%)	0.333
No	2 (7.41%)	7 (25.00%)		5 (25.00%)	10 (20.83%)		0 (0.00%)	5 (13.16%)		7 (13.21%)	22 (19.30%)	

*VAT* Visceral adipose tissue, *SAT* subcutaneous adipose tissue, *MFI* mesenteric fat index, *VAT* area/FM the ratio of visceral adipose tissue to fat mass, *BMI* body mass index

*Underweight, BMI < 18.5 kg/m^2^; Normal BMI, 18.5 ≤ BMI < 24.0; Overweight, 24.0 ≤ BMI < 28.0

**Some patients with missing biological data were not analyzed

For Group A, compared with all patients, the incidence of recurrence within 1 year in patients received 5-amino salicylic acid treatment was also related with higher erythrocyte sedimentation rate (ESR) and C-reactive protein (CRP) than baseline. Patients with normal ESR (*P* = 0.039) and normal CRP (*P* = 0.002) value were more likely to not relapse. There was no statistical difference in MFI in Group B and no factors were associated with relapse in Group C.

**Fig. 3:**
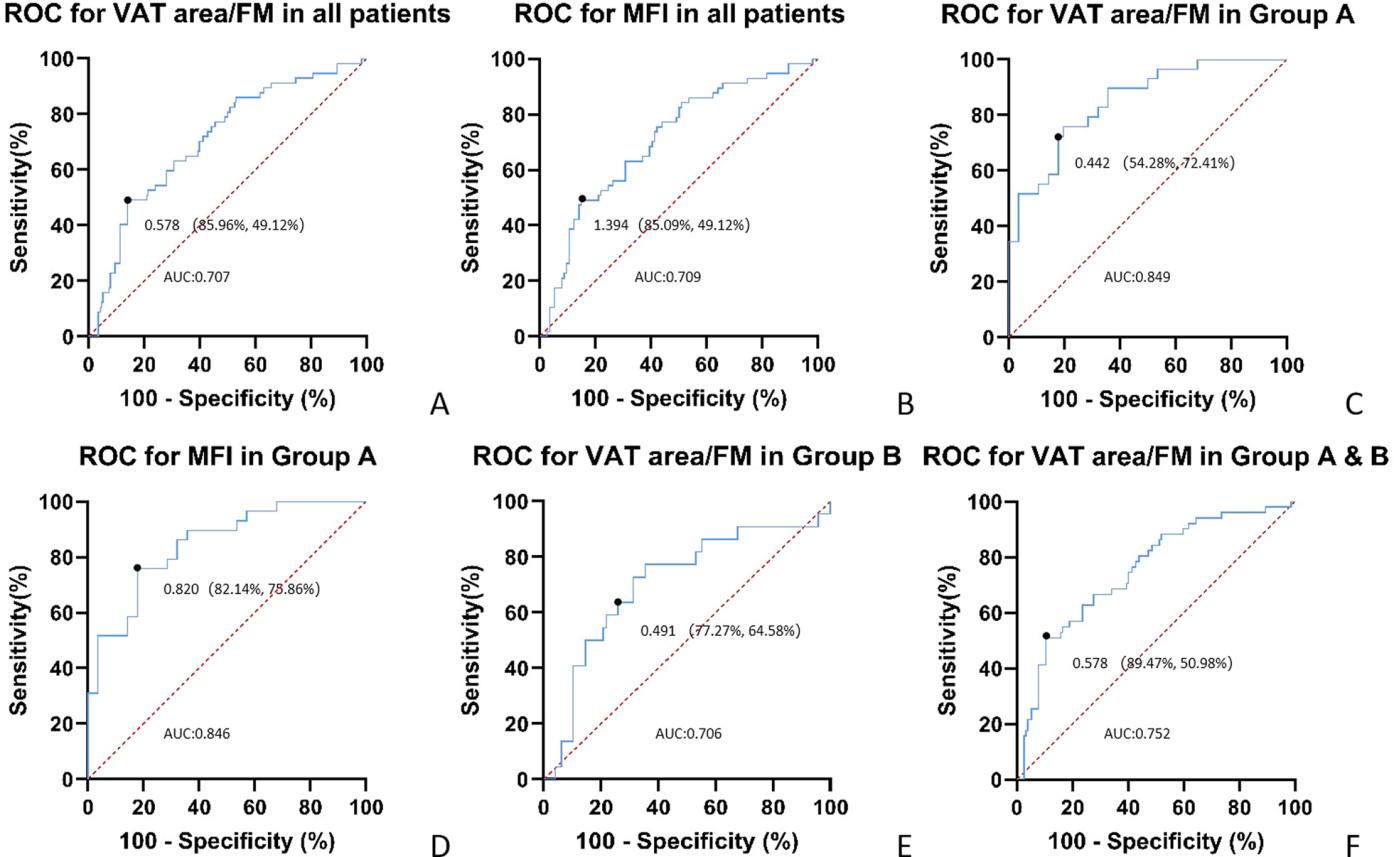
The ROC of the VAT area/FM with significant differences in all patients and in Group A, Group B and Group A & B, the ROC of the MFI with significant differences in all patients and Group A. The AUC of the VAT area/FM in all patients’ curve with 49.12% sensitivity and 85.96% specificity was 0.707 [95% CI 0.625–0.789]. Patients with a ratio higher than 0.578 tended to be recurrence within 1 year (**A**). The AUC of the MFI in all patients’ curve with 49.12% sensitivity and 85.09% specificity was 0.709 [95% CI 0.627–0.791]. Patients with a value higher than 1.394 tended to be recurrence within 1 year (**B**). The AUC of the VAT area/FM in Group A curve with 72.41% sensitivity and 54.28% specificity was 0.849 [95% CI 0.752–0.950]. Patients with a ratio higher than 0.442 tended to get recurrence within 1 year (**C**). The AUC of the MFI in Group B curve with 75.86% sensitivity and 82.14% specificity was 0.820 [95% CI 0.748–0.945]. Patients with a value higher than 1.319 tended to get recurrence within 1 year (**D**). The AUC of the VAT area/FM in Group B curve with 64.58% sensitivity and 77.27% specificity was 0.706 [95% CI 0.568–0.844]. Patients with a ratio higher than 0.491 tended to get recurrence within 1 year (**E**). The AUC of the VAT area/FM in Group A & B curve with 50.98% sensitivity and 89.47% specificity was 0.752 [95% CI 0.665–0.838]. Patients with a ratio higher than 0.578 tended to get recurrence within 1 year (**F**). Since there was no significant difference between VAT area/FM or MFI and whether recurrence within 1 year or not in other groups, the ROC curve was not drawn

Figure 3 showed the performance of VAT area/FM and MFI to distinguish whether patients would relapse within 1 year. Patients with higher than 0.578 VAT area/ FM or higher than 1.394 MFI had higher chance of recurrence within 1 year, with AUC of 0.707 [95% CI 0.625–0.789] and 0.709 [95% CI 0.627–0.791]. The ROC curve demonstrated that the VAT area/FM could differentiate recurrence from non-recurrence efficiently in Group A, with an AUC of 0.849 [95% CI 0.752–0.950].

Table 3 showed the result of the LASSO and the coefficient of the enrolled factors. Since no factors were enrolled when the Mean square of error (MSE) met the minimum in Group B and C in the building process of LASSO, the result was not listed. A total of 2 factors were enrolled in the process of model building in all patients including VAT area/FM and sarcopenia with 0.20 MSE. A total of 3 factors were enrolled in Group A with 0.14 MSE. The most significant factor was VAT area/FM with a 1.55 coefficient.

**Table 3:** The coefficients of variables in Group A and overall

Variable*	Coefficients	Lambda	MSE
*Group A*			
VAT area/FM	1.55	0.06	0.14
High CRP	0.26		
High ESR	0.03		
*Overall*			
VAT area/FM	0.47	0.06	0.20
Sarcopenia	0.03		

*The MSE was minimum when none variables were enrolled in Group B and C

## Discussion

To the best of our knowledge, this is the first study focusing on the relationship between radiological data and the recurrence incidence within 1 year under different treatments of newly diagnosed CD. We found that for all enrolled patients, MFI, VAT area/FM, and diagnosis of sarcopenia were three radiological factors related to relapsing within 1 year. ROC curve demonstrated that patients with the ratio of VAT area/FM and MFI higher than 0.578 and 1.394 tended to relapse with the AUC of 0.707 and 0.709. Albumin was the only biological factor related to the recurrence.

Visceral adipose tissue (VAT) refers to the white adipose tissue that surrounds the viscera was demonstrated to play a key role in inflammation regulation [23]. VAT accumulation can be seen in most small bowel resection specimens of early CD patients, but not in other gastrointestinal diseases [24]. In addition, VAT was associated the occurrence of ulcers, strictures, intestinal wall thickening and transmural inflammation in CD [25]. Therefore, the content of VAT may have a have a potential role to reflect the severity and activity of CD and predict the prognosis of patients. In our study, AT area/FM and MFI (the ratio of VAT area to SAT) were closely associated with the disease recurrence rather than VAT area. This may be explained by the significant variation of the absolute content of VAT among different patients, or the function of SAT as a potential protective factor for CD. In previous studies, MFI and VAT area/FM were two risk factors of penetrating lesions and structuring lesions [26, 27]. Therefore, it is understandable that these two factors are related to 1-year recurrence of CD. The SM and SMI were not predictive factors for recurrence. Sarcopenia, on the contrary, was related to the recurrence, which was also demonstrated in previous study. The reason for this result is not clear. Holt et al. found that inflammatory biomarkers showed an inverse correlation with skeletal muscle mass. This may relate to the calprotectin caused by the higher serum [28]. Moreover, SM plays an anti-inflammatory effect through the IL-6, IL-7 and IL-15 [29, 30].

Although sarcopenia was a predictive factor for recurrence in all patients, there was no significant difference in each group. This indicated that sarcopenia was a false positive factor. Besides, the visceral obesity was not a factor for recurrence either. This finding was not exactly consistent with the previous studies [12, 14, 15]. This could be explained by two reasons. First, the end-point for follow-up was different. In the studies of Claire et al. [15] and Julienne et al. [14], the adverse outcomes were defined as surgery or death because of the disease progression. In our study, however, whether patients obtained recurrence within 1 year or not based on the SES-CD score was the endpoint for follow-up. Another important reason is that the impact of different treatment was not considered in their studies. Although the MFI, VAT area/FM and sarcopenia were related to the recurrence in patients treated with 5-amino salicylic acid (Group A), this result could be obtained from patients treated with corticosteroids and immunosuppressants (Group B) and biologics (Group C). This shows that the topper strategy could reduce the effect of VAT on disease progression and reduce the recurrence rate.

The metabolite of immunosuppressants such as azathioprine was 6-thioguanine nucleotide (6-TGN) which are related to the effect of CD remission [31]. Holt et al. demonstrated that VAT area and SAT area had no association the level of 6-TGN [32]. This may explain the reason why MFI was not related to the recurrence. An interesting finding was that the content of VAT did not seem to have a significant effect on the biologics, since VAT was not related to the recurrence rate in Group C. This is not consistent with the previous study, which indicated that VAT was independently related to the weakening of mucosal healing in the use of infliximab [16]. It should be pointed out that patients in Group C were younger than patients in Group A, but we believe that the main reason for the relevance of VAT to the recurrence of Group A patients and the irrelevance of VAT to the recurrence of Group C patients was the treatment rather than age. This is because previous studies showed that the content of VAT increases with age [33–35] and the accumulation of visceral adipose tissue would lead to recurrence or complications of CD patients [26, 27]. As a result, without considering the impact of treatment, older patients had higher risk of CD recurrence or serious complications. However, this conclusion is contradictory to the current study [36]. Consequently, the reason for a lack of difference in visceral fat indices between recurrence/non-recurrence is more likely to be the biologics instead of age. Furthermore, in order to better verify this speculation, we are currently conducting a prospective cohort study to determine the relationship between recurrence in patients treated with biologics and VAT.

In conclusion, for patients with high VAT area/FM and MFI tended to have recurrence if they were treated with 5-amino salicylic or azathioprine + steroids. The radiological data did not seem to have relevance with the endoscopic recurrence for patients treated with biologics. This indicated that biologics could be a protective factor of high VAT area/FM and MFI. Moreover, according to the Chinese CD guideline, CD patients with more than two high risk factors among which VAT and MFI are not included are more recommended to use biologics [4]. Therefore, we suggested that these two factors should be added to the guideline. The use of biologics may be used for those patients with high VAT area/FM and MFI to avoid the recurrence within 1 year.

This study has a few limitations. First, this is a single-center retrospective study with a small sample size. Second, we did not include patients undergoing surgery in this study, since surgical patients will continue to use different medications after surgery, which will greatly interfere the results of the study. Third, the duration of follow-up is not long. The lack of positive results in the biologics group may be related to the duration of follow-up, which has lasted only 1 year. Therefore, a longer-term multi-center study with a lager simple size and longer-term study is needed.

## Conclusion

High VAT area/FM and MFI had relevance with 1-year recurrence for newly diagnosed CD patients treated by 5-amino salicylic or azathioprine + steroids while they had no impact on the recurrence of patients treated with biologics. No radiological data were related to the recurrence for patients treated with biologics.

## Data Availability

Data generated by and used in the study is available from the corresponding author upon reasonable request.

## Abbreviations

CD: Crohn’s Disease
CDAI: Clinical Disease Activity Index
CRP: C-reactive Protein
CT: Computed Tomography
ESR: Erythrocyte Sedimentation Rate
FM: Fat Mass
HU: Hounsfield Unit
MFI: Mesenteric Fat Index
MSE: Mean Square of Error
ROC: Receiver Operating Characteristic
SAT: Subcutaneous Adipose Tissue
SES-CD: Simplified Endoscopic Activity Score for Crohn’s Disease
SM: Skeletal Muscle
VAT: Visceral Adipose Tissue
6-TGN: 6-Thioguanine Nucleotide
